# Exploratory factor and confirmatory analyses of the polycystic ovary syndrome health-related quality of life questionnaire (PCOSQ-50)

**DOI:** 10.1186/s12955-024-02228-z

**Published:** 2024-02-03

**Authors:** Pamela J. Wright, Abbas S. Tavakoli, Robin M. Dawson

**Affiliations:** 1 College of Nursing, University of South Carolina, Columbia, SC USA; 2 Advancing Chronic Care Outcomes through Research and iNnovation (ACORN) Center, College of Nursing, University of South Carolina, Columbia, SC USA

**Keywords:** Polycystic ovary syndrome, Health-related quality of life, Statistical factor analysis, Reproducibility of results

## Abstract

**Background:**

A condition-specific instrument is necessary to measure the health-related quality of life among those with polycystic ovary syndrome (PCOS), the most common chronic endocrinopathy among women. The first instrument was developed in 1988, followed by several revisions. However, further recommendations from all versions include additional application and measurement among different cultural populations of women with PCOS and psychometric testing based on use among larger samples of women with PCOS. Thus, the purpose of this study was to explore the factor structure of the Polycystic Ovary Syndrome Questionnaire (PCOSQ-50) using an international cross-sectional survey data from women with PCOS aged 18–42 years.

**Methods:**

Using data from the largest known international cross-sectional study of women with PCOS aged 18–42 years (*n* = 935) to date, exploratory factor and confirmatory analyses were conducted for the PCOSQ-50, followed by factor labeling using a thematic analysis approach.

**Results:**

Respondents were 31.0 ± 5.8 years of age, mostly White (72%), well-educated (56% had a college degree), married (69%), and employed full-time (65%). Three-quarters (74%) of the sample had one or more chronic conditions in addition to PCOS. Approximately 20% of the respondents originated from countries such as the United Kingdom, Australia, South Africa, etc. The PCOSQ-50 demonstrated good reliability but may be best described using a 7-factor model. The 7-factor model revealed goodness-of-fit. Thematic analysis suggested the following labels of those seven factors: hirsutism, fertility, isolation/trepidation, sexual function, self-esteem, emotional, and obesity.

**Conclusion:**

More research is needed to adapt the current PCOSQ-50, as well as to create an age-appropriate PCOS-specific HRQoL instrument for peri-postmenopausal women with PCOS.

**Supplementary Information:**

The online version contains supplementary material available at 10.1186/s12955-024-02228-z.

## Background

Health-related quality-of-life (HRQoL) has been defined as the physical, psychological, and social domains of health, seen as distinct areas that are influenced by a person’s experiences, beliefs, expectations, and perceptions [1]. Thus, HRQoL is a multi-dimensional concept commonly used to examine the impact of the presence and treatment of chronic health conditions on an individual’s physical, emotional, and social well-being [2]. Several generic instruments, such as the 36 Item Short-Form (SF-36®) Survey [3], were developed to measure HRQoL using patients’ commonly reported outcomes. Whereas generic HRQoL instruments can be used with most any health condition, they lack specificity for certain health conditions, including polycystic ovary syndrome (PCOS). PCOS, the most common endocrinopathy among women [4], presents with complex signs and symptoms (e.g., subfertility, hirsutism) of hormonal dysregulation that negatively impact HRQoL [5]. Thus, PCOS-specific HRQoL instruments are necessary to assess HRQoL more accurately [5] among the approximately 20 million [4] women with this chronic health condition.

In 1988, Cronin and colleagues created the first PCOS-specific HRQoL instrument by interviewing a clinical population of women with PCOS aged 18–45 years (*n* = 100) to identify issues associated with PCOS [6]. The final choice of questions was based on the authors’ “clinical sensibility” and factor analysis. This instrument, the original Polycystic Ovary Syndrome Questionnaire (PCOSQ) has 26 items organized in five domains: emotions (8 items), body hair (5 items), weight (5 items), infertility (4 items), and menstrual problems (4 items). Each item is answered using a 7-point Likert scale, with 7 representing optimal function and 1 representing the poorest function [6]. As knowledge advanced about PCOS and its effect on HRQoL, researchers from the United Kingdom sought to validate the PCOSQ by determining its factor structure [7]. The PCOSQ was modified by adding four additional questions about acne and separating the domain of menstrual problems into two domains: menstrual symptoms and menstrual predictability. The 7-point Likert scale was retained. However, the psychometrics of both the PCOSQ and the modified PCOSQ (MPCOSQ) revealed poor face and content validity indices, with low alpha coefficients for the domains of menstrual problems (0.56) and emotions (0.60) [8].

Based on the poor psychometrics of the PCOSQ and MPCOSQ, Nasiri-Amiri and colleagues (2016) conducted a mixed-method, sequential, exploratory design to define the components of PCOS-specific HRQoL, develop a more comprehensive instrument to assess PCOS-specific HRQoL among Iranian women aged 18–40 years, and assess its psychometric properties [9]. The new instrument included 50 items in six domains: psychosocial/emotional, fertility, sexual function, obesity/menstrual disorders, hirsutism, and coping and is referred to as the PCOSQ-50. Items within each domain are answered using a 5-point Likert scale, with 1 representing the worst condition and 5 representing the best condition. Assessment of the psychometric properties of the PCOSQ-50 revealed a mean content validity index and ratio of 0.92 and 0.91, respectively, a Cronbach’s alpha of 0.88, Spearman’s correlation coefficients of test–retest of 0.75, and an intra-class correlation coefficient for the subscales ranging from 0.57 to 0.88 [9]. Stevanovic and team (2018) found similar psychometric properties for the PCOSQ-50 when using and assessing the instrument among a small sample of Serbian women [10].

In 2018, Nasiri-Amiri and associates performed exploratory factor analysis and confirmatory analysis to further examine the factor structure of the PCOSQ-50. Based on results, 6 items were omitted, and the coping domain was replaced with a body image domain. The revised version, the PCOSQ-43, had a Cronbach’s alpha of 0.92 and an intra-class correlation coefficient that ranged from 0.91 to 0.94. Thus, the research team concluded that the PCOSQ-43 showed marked improvement in reliability [11]. Despite these improvements, acceptance and usability of this version remains unexplored, as the PCOSQ-26 and the PCOSQ-50 are the more commonly applied PCOS-specific HRQoL instruments. To date, further recommendations of all versions include additional application and measurement among different cultural populations of women with PCOS and psychometric testing based on use among larger samples of women with PCOS [9–12].

In addition to this call for further testing, a primary impetus for exploratory factor and confirmatory analyses stemmed from the cross-sectional study (*n* = 935) used for this study [13]. Many women (~ 50) replied to the first author (PJW) via social media messaging, reporting “offense” to the wording of questions in the current instrument, as well as a feeling of exclusivity due to a perceived focus on reproduction. Thus, the purpose of this study was to explore the factor structure of the PCOSQ-50 using an international cross-sectional survey data from women with PCOS aged 18–42 years.

## Methods

### Study design and participants

A cross-sectional study design was used to describe the HRQoL of women with PCOS aged 18–42 (*n* = 935). The study participants were recruited from two PCOS-specific Facebook groups. Inclusion criteria were women who self-reported a PCOS diagnosis. If eligible, women were invited to complete a cross-sectional internet-based survey using Research Electronic Data Capture (REDCap) to assess PCOS-specific HRQoL. An electronic link led potential participants to a website that provided additional details about the study. The introductory description of the study allowed the women to make an informed decision about participating. Participants were informed that completing the survey would constitute implied consent. *The Completely Automated Public Turing test to tell Computers and Humans Apart* (CAPTCHA) was used to minimize non-human responses (e.g., robotic [“bot”], spam). Participants had the option to enter a drawing to win one of twelve US $50 gift cards. In accordance with 45 CFR 46.104(d)(2) and 45 CFR 46.111(a)(7), the University of South Carolina (USC) Institutional Review Board provided an “exempt” status for the study (Pro00118636) because the research involved surveys in a manner that the identity of respondents could not be readily ascertained [13].

### Facebook groups

The two PCOS-specific Facebook pages used to post the survey link were titled *PCOS Support Group* (21,200 members) and *PCOS Diet Support* (18,000 members). Members of each Facebook page were required to apply for membership, which helped to protect against robotic responses. As these groups were private and required administrator approval for membership, the PI contacted the administrators of each group to explain the study and address any concerns. The administrators then posted the survey link on the message board, thus allowing members to access the survey.

### Measures

#### Demographics

The demographic questionnaire included age, race, geographic location, educational attainment, number of children and comorbid conditions, and marital, employment and insurance status. Respondents self-reported a diagnosis of PCOS. Based on studies examining concordance between self-report and medical diagnoses, self-report has good concordance with electronic medical records and greater than 90% specificity for all medical diagnoses [14, 15].

#### PCOS-specific HRQoL

PCOS-specific HRQoL was measured using the PCOSQ-50. It is a PCOS-specific HRQoL instrument that includes 50 questions representing women’s perceptions of symptom severity across six domains: psychosocial/emotional, fertility, sexual function, obesity/menstrual disorders, hirsutism, and coping. Responses to all items are rated on a 5-point Likert-type scale ranging from 0 = never (best condition) to 4 = always (worst condition). Each domain results in a subscale score that is calculated as the sum of all answered items divided by the number of answered items in that domain. The total PCOSQ-50 score is calculated as the sum of all answered items divided by the number of answered items. Per the PCOSQ-50 scoring guidelines, missing items are not included when calculating the domain subscale scores or the total PCOSQ-50 score. Lower scores indicate a better HRQoL. Construct validity was reported at 0.92 and test–retest reliability was reported at 0.91 [11].

### Data analysis

#### Dataset

There were no missing data. The dataset met the assumption of normality with a skewness of − 0.05 and kurtosis of 1.0.

#### Exploratory factor analysis

Exploratory factor analysis (EFA) was run for the PCOSQ-50. EFA was conducted using squared multiple correlations as prior communality estimates. The maximum Likelihood (ML) method was used to extract the factors followed by the promax (oblique) rotation. Factor loadings were assessed using item communalities, cross-loadings, and item statistics. Parallel analysis was conducted to examine the number of factors to extract. Parallel analysis produces correlation matrices from a randomly chosen simulated dataset that has a similar number of observations as the original dataset [16]. The number of factors to be retained from comparing simulated and original datasets to determine the point at which the eigenvalue in the simulated data was higher than the original data. In addition, the scree plot was examined to verify factor retention. The number of items loading onto a given factor determined the strength of a factor. In interpreting the rotated factor pattern, an item was said to load on a given factor if the factor loading was 0.35 or greater for that factor and was less than 0.35 for the other. Fit indices used in this study included normed chi-square (X^2^/df), Kaiser-Meyer-Olkin (KMO), and Root Mean Square Residual (RMSR). Model fit criteria were a normed chi-square of less than 3, RMSEA between 0.05 and 0.08, and KMO values smaller than 0.50 indicates that factor analysis is not suitable [17], values between 0.50 and 0.70 are considered average, values between 0.70 and 0.80 are good and values between 0.90 and 1.00 are excellent [18].

#### Confirmatory analysis

Confirmatory factor analysis (CFA) was performed to validate the factors associated with the PCOSQ-50. Goodness-of-fit for CFA included chi-square statistics, ratio of chi-square and degree freedom, the normed fit index (NFI), the non-normed fit index (NNFI), the comparative fit index (CFI), root mean squared error (RMSE), and standardized root mean square residual (SRMSER). The range for NFI, NNFI, and CFI were between 0.00 to 1.00. A value close to 1.00 represents a good fit of model. The model is a good fit when RMSEA is between 0.05 and 0.08 [19].

All data analyses were performed using SAS statistical software, version 9.4 [20].

#### Labeling the domains

After identification of the factor model, our next step was to analyze the factor groupings and assign descriptive names, thus creating topic areas called domains or subscales for the PCOSQ-50. Two researchers (PJW, RMD) accomplished this by using a thematic analysis approach, focusing on commonalities among questions within each factor grouping and identifying key words in each question [21]. PJW (a nurse scientist with experience working with women with PCOS) and RMD (a qualitative methodologist and nurse practitioner familiar with PCOS medical management) independently coded each factor grouping, then met to collaboratively discuss and reconcile minor differences between codes. Subsequently, they followed an iterative categorization process to draw connections between the codes, which were subsequently organized into themes. Rigor was strengthened through reflexivity activities of the two researchers, including regular meetings to discuss personal experiences with and clinical knowledge of PCOS. Continuous reassessment and reiteration of coding further strengthened rigor.

## Results

Respondents (*n* = 935) were 31.0 ± 5.8 years of age, mostly White (72%), well-educated (56% had a college degree), married (69%), and employed full-time (65%). Nearly three-quarters (74%) of the sample had one or more chronic conditions in addition to PCOS, such as hypertension, type 2 diabetes, arthritis, and hypothyroidism. Using social media allowed participation from within and outside the US: 80% of the respondents in the sample were from the US. The geographic areas and the number of respondents from each region are detailed in an additional file (see Additional file 1). See Table 1.

**Table 1:** Demographic and health-related characteristics of the women with PCOS aged 18–42 (*n* = 935)

Variable	*n* = 935	
	#	%
**Race**		
African American/Black	230	24.6
American Indian/Native American	10	1.1
Asian	54	5.8
Latino	77	8.2
Middle Eastern/N African	4	0.4
White	515	55.1
Mix of Two	34	3.6
Prefer Not to Answer	11	1.2
**Educational Attainment**		
Some High School	14	1.5
High School or GED	69	7.7
Some College	313	34.8
Bachelors	312	34.7
Masters	158	17.6
Doctorate	26	2.9
Prefer Not to Answer	7	0.8
**Employment Status**		
Not Working	166	18.7
Part-Time	127	14.3
Full-Time	588	66.2
Prefer Not to Answer	7	0.8
**Medical Insurance**		
Yes	775	86.1
No	109	12.1
Prefer Not to Answer	16	1.8
**Marital Status**		
Single	250	28.0
Married/Partnership	616	69.0
Divorced	25	2.8
Widowed	0	0.0
Prefer Not to Answer	2	0.2
**# Children**		
0	512	55.0
1–2	303	32.0
3–4	75	8.0
≥ 5	4	0.4
Prefer Not to Answer	41	4.6
**# Comorbid Conditions**		
0	240	27.1
1–2	494	55.7
3–4	153	17.2
≥ 5	0	0.0

The means and standard deviations were calculated for the total HRQoL and HRQoL subscales and displayed in Table 2.

**Table 2:** HRQoL total score and HRQoL subscale scores of women with PCOS (*n* = 935)

Variable	Mean	SD
HRQoL Total*	**2.52**	**0.96**
Psychosocial/Emotional	2.59	0.67
Fertility	3.15	1.10
Sexual Function	1.82	1.04
Obesity/Menstrual	2.59	0.75
Hirsutism	2.60	1.36
Coping	2.37	0.85

*A lower score on the HRQoL scale and each subscale (range 0.00–4.00) indicates a better HRQoL

Table 3 shows the means and standard deviations (SD) for the questions in each of the six subscales. The lowest average was 1.36 with SD of 1.41 for the fertility item PCOSQB7 (In the past 4 weeks, how often have you experienced fear of divorce or separation?) and the highest average was 3.27 with SD of 1.23 for the obesity/menstrual item PCOSD1 (In the past 4 weeks, how often have you felt concerned about being overweight?). Table 3 details the means and standard deviations for all questions in each of the six subscales.

**Table 3:** Means and standard deviations of every item on the PCOSQ-50 (*n* = 935)

Item	Description	mean	std
**Psychosocial/Emotional**			
pcosq1	In the past 4 weeks, how often have you suffered from bad mood due to PCOS?	2.48	0.86
pcosq2	In the past 4 weeks, how often have you experienced impatience due to PCOS?	2.54	0.98
pcosq3	In the past 4 weeks, how often have you blamed yourself for having PCOS?	2.01	1.46
pcosq4	In the past 4 weeks, how often have you experienced trouble dealing with others?	2.15	0.90
pcosq5	In the past 4 weeks, how often have you suffered from low self-esteem due to PCOS?	3.02	1.03
pcosq6	In the past 4 weeks, how often have you experienced aggressiveness due to PCOS?	1.82	1.09
pcosq7	In the past 4 weeks, how often have you felt pessimistic about the treatment?	2.57	1.11
pcosq8	In the past 4 weeks, how often have you suffered from the embarrassment due to your appearance?	2.83	1.13
pcosq9	In the past 4 weeks, how often have you felt different to normal women?	3.01	1.13
pcosq10	In the past 4 weeks, how often have you experienced lack of control of emotions?	2.38	0.98
pcosq11	In the past 4 weeks, how often have you felt ugly or unattractive?	2.99	1.03
pcosq12	In the past 4 weeks, how often have you felt easily tired?	3.31	0.81
**Fertility**			
pcosqb1	In the past 4 weeks, how often have you felt sad seeing children?	1.64	1.43
pcosqb2	In the past 4 weeks, how often have you felt sad seeing pregnant women?	1.89	1.56
pcosqb3	In the past 4 weeks, how often have you experienced concern about infertility?	2.28	1.58
pcosqb4	In the past 4 weeks, how often have you felt you will accept all other PCOS manifestations if assured of pregnancy?	1.82	1.51
pcosqb5	In the past 4 weeks, how often have you felt fear of abortion?	1.74	1.57
pcosqb6	In the past 4 weeks, how often have you felt concerned about infertility in the future?	2.48	1.55
pcosqb7	In the past 4 weeks, how often have you experienced fear of divorce or separation?	1.17	1.31
pcosqb8	In the past 4 weeks, how often have you felt uselessness of sexual intercourse due to infertility	1.36	1.40
pcosqb9	In the past 4 weeks, how often have you experienced concern about the long term effects of PCOS medication?	2.26	1.34
**Sexual Function**			
pcosqc1	In the past 4 weeks, how often have you felt unsatisfied with sex?	1.84	1.31
pcosqc2	In the past 4 weeks, how often have you experienced lack of sexual stimulation?	1.99	1.28
pcosqc3	In the past 4 weeks, how often have you experienced lack of sexual desire?	2.17	1.26
pcosqc4	In the past 4 weeks, how often have you experienced lack of lubrication during sexual intercourse?	1.51	1.32
pcosqc5	In the past 4 weeks, how often have you experienced lack of orgasm?	1.70	1.31
pcosqc6	In the past 4 weeks, how often have you felt ashamed of sexual coldness/unresponsiveness?	1.73	1.43
pcosqc7	In the past 4 weeks, how often have you experienced lack of libido because of PCOS?	1.96	1.36
**Obesity/Menstrual**			
pcosqd1	In the past 4 weeks, how often have you felt concerned about being overweight?	3.52	0.93
pcosqd2	In the past 4 weeks, how often have you felt the need to decrease your weight to control PCOS status?	3.49	0.94
pcosqd3	In the past 4 weeks, how often have you felt concerned about a fast return to your previous weight after any weight loss?	3.12	1.18
pcosqd4	In the past 4 weeks, how often have you felt concerned about the complete cessation of menstruation?	2.03	1.46
pcosqd5	In the past 4 weeks, how often have you felt concerned about menstruation at long intervals?	1.90	1.45
pcosqd6	In the past 4 weeks, how often have you felt willingness to reduce your weight to be more attractive for your spouse?	2.92	1.26
pcosqd7	In the past 4 weeks, how often have you experienced fear of diseases such as diabetes, hypertension, and heart disease?	2.73	1.27
pcosqd8	In the past 4 weeks, how often have you felt the urge to abandon treatments because of repetitive visits to doctors?	1.78	1.40
pcosqd9	In the past 4 weeks, how often have you experienced fear of cancer?	1.86	1.35
**Hirsutism**			
pcosqe1	In the past 4 weeks, how often have you felt embarrassed because of excess facial hair?	2.68	1.45
pcosqe2	In the past 4 weeks, how often have you felt concerned about the progression of excess body and facial hair?	2.71	1.40
pcosqe3	In the past 4 weeks, how often have you felt concerned about having excess facial hair?	2.68	1.45
pcosqe4	In the past 4 weeks, how often have you felt concerned about rapid regrowth of unwanted hair after its removal?	2.66	1.43
pcosqe5	In the past 4 weeks, how often have you felt embarrassed because of having excess body hair?	2.63	1.44
pcosqe6	In the past 4 weeks, how often have you felt the need to cover your body and face because of excess hair?	2.26	1.56
**Coping**			
pcosqf1	In the past 4 weeks, how often have you felt a lack of family support and acceptance of your disease?	1.86	1.39
pcosqf2	In the past 4 weeks, how often have you felt a lack of satisfaction with being a woman?	2.10	1.34
pcosqf3	In the past 4 weeks, how often have you felt the desperate need for a cure?	3.13	1.08
pcosqf4	In the past 4 weeks, how often have you felt the need to complain with others about PCOS?	2.08	1.20
pcosqf5	In the past 4 weeks, how often have you felt lack of satisfaction with your appearance (self image)?	1.91	1.36
pcosqf6	In the past 4 weeks, how often have you felt lack of satisfaction with your role/future role as a wife?	3.23	0.95
pcosqf7	In the past 4 weeks, how often have you felt lack of satisfaction with your role as a spouse or partner?	2.24	1.36

There were no missing values for any items

Model fit criteria were a normed chi-square of close to three for 7 factors (Table 4). The Kaiser-Meyer-Olkin (KMO) measure of sampling adequacy is 0.93, which is acceptable. The residuals are all small and the overall Root Mean Square Residual (RMSR) is 0.05,0.04, and 0.03 for four factors, five factors, and six/seven factors; respectively, indicating that the factor structure explains most of the correlations (Table 4).

**Table 4:** Model fit and the Kaiser-Meyer-Olkin (KMO) for the PCOSQ-50 (*n* = 935)

Models Tested	Model Chi Square	∆ χ^2^	χ^2^ /df	RMSA	KMO
**4 Factor**	6075.1	NA	5.89	0.05	0.93
**5 Factors**	4693.6	1381.5	4.76	0.04	0.93
**6 Factors**	3750.7	942.9	3.99	0.03	0.93
**7 Factors**	2985.8	764.9	3.33	0.03	0.93

**Fig. 1:**
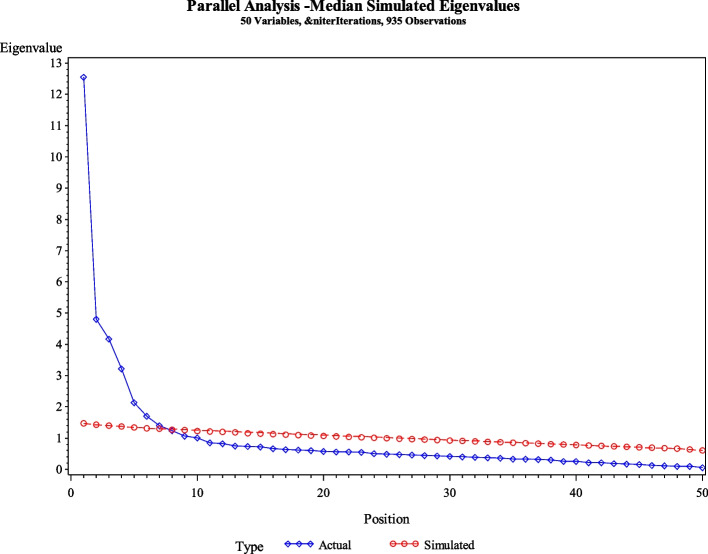
Parallel analysis for the PCOSQ-50 (*n* = 935)

**Fig. 2:**
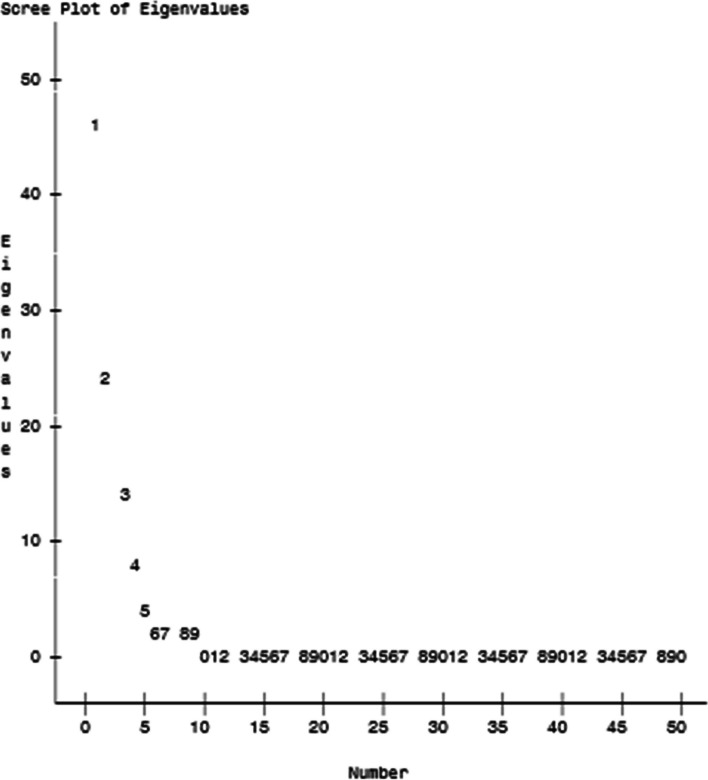
Scree plot of eigenvalues for the PCOSQ-50 (*n* = 935)

Table 5 reports the rotated Factor Pattern (Standardized Regression Coefficients) for the items in each subscale of the PCOSQ-50. Parallel analysis indicated four or five factors should be retained (Fig. 1). A Scree plot of eigenvalues greater than one (Fig. 2) and the proportion of variance explained 90% for 4 factors, 95% for 5 factors, 97% for 6 factors, and 99% for seven factors (each factor presented 4, 5, 6, or 7 meaningful factors for the 50-item scale). Items pcosqf1, pcosqf2, pcosqf7, pcosqf5, pcosqf4, pcosqb9, and pcosqd9 did not load for any of the factors for the four-factor model. Items pcosq5, pcosq8, and pcosq11 were loaded for more than one factor in the six-factor model.

**Table 5:** Factor loading (standardized regression coefficients) of the PCOSQ-50 (*n* = 935)

Items	4 Factor				5 Factor					6 Factor						7 Factor						
	Fac 1	Fac 2	Fac 3	Fac 4	Fac 1	Fac 2	Fac 3	Fac 4	Fac 5	Fac 1	Fac 2	Fac 3	Fac 4	Fac 5	Fac 6	Fac 1	Fac 2	Fac 3	Fac 4	Fac 5	Fac 6	Fac 7
pcosq5	76				60									63						66		
pcosq11	76				50									93						95		
pcosqf6	71								42					69						72		
pcosq8	67				41									70						72		
pcosqd6	65								53						39							37
pcosqd1	62								53						83							82
pcosq1	62				78					73											76	
pcosqd2	60								91						88							88
pcosqf2	54				55					38								50				
pcosqf3	52				44					40								61				
pcosq4	51				68					67											66	
pcosq10	51				75					69											59	
pcosq2	51				71					76											76	
pcosqf1	51				50					47								53				
pcosqd3	50								59						60							56
pcosq3	49				49					30										30		
pcosq6	48				70					79											74	
pcosq7	47				43					29								30				
pcosqf4	46				46					48								56				
pcosq9	46				37									31						32		
pcosqf5	46				44					47								52				
pcosqf7	43				45					34								35				
pcosqd7	40								29						29			48				
pcosq12	39				39					32											29	
pcosqd8	38				39					42								58				
pcosqb9	36				30					35								52				
pcosqd9	36									39								59				
pcosqb7	33				43													33				
pcosqd5	29									32								40				
pcosqb3		96				96					97						97					
pcosqb6		92				93					92						93					
pcosqb2		84				84					86						86					
pcosqb4		81				81					81						80					
pcosqb1		78				77					78						78					
pcosqb5		77				76					76						74					
pcosqb8		56				54					54						53					
pcosqd4		36				35					34							38				
pcosqe3			98				98					98				99						
pcosqe1			96				97					96				97						
pcosqe2			94				94					94				94						
pcosqe5			91				91					91				91						
pcosqe4			91				91					91				91						
pcosqe6			80				81					80				80						
pcosqc3				90				91					91						92			
pcosqc7				87				88					88						88			
pcosqc2				85				84					85						84			
pcosqc6				77				76					77						77			
pcosqc1				65				65					65						65			
pcosqc4				56				56					56						56			
pcosqc5				55				54					54						53			

Root Mean Square Residual (RMSR) = .05 for 4 factors, .04 for 5 factors, .03 for 6 factors, and .03 for 7 factors

Kaiser’s Measure of Sampling Adequacy = .93

Eigen Values: Fact1 = 45.37, Fact2 = 23.74, Fact3 = 13.73, Fact4 = 8.67, Fact5 = 4.10, Fact6 = 2.53, Fact7 = 2.02

Item did not load: pcosqd5 (for 5 factors)

Tables 6, 7, 8 and 9 show the intercorrelations and the coefficient alpha reliability. Scale reliability was assessed by calculating a coefficient alpha. Reliability estimates were shown in the diagonal. The results revealed that each reliability exceeded 0.75. The alpha coefficient ranged from 0.89 to 0.97 for 4-factors, 0.86 to 0.97 for 5-factors, 0.87 to 0.97 for 6-factors, and 0.83 to 0.97 for 7-factors. The results indicated that all the correlations are significant among these total scales and subscales. All correlations were positive and range from 0.11 to 0.78 for 4-factors, 0.08 to 0.85 for 5-factors, 0.08 to 0.86 for 6-facotrs, and 0.05 to 0.81 for 7-factors.

**Table 6:** Means, standard deviations, pearson correlations, and reliabilities for the total scale and six subscales of the PCOSQ-50 for four factors (*n* = 935)

Factors	Mean	SD	Total	Factor 1	Factor2	Factor 3	Factor 4
Total	116.17	31.41	0.93	0.91	0.65	0.55	0.50
				<.0001	<.0001	<.0001	<.0001
				0.92			
Factor 1	72.43	18.73			0.46	0.39	0.32
					<.0001	<.0001	<.0001
					0.92		
Factor 2	15.25	9.64				0.13	0.14
						<.0001	<.0001
						0.97	
Factor 3	15.61	8.14					0.11
							.0008
							0.89
Factor 4	12.89	7.26					–

**Table 7:** Means, standard deviations, pearson correlations, and reliabilities for the total scale and six subscales of the PCOSQ-50 for five factors (*n* = 935)

Factors	Mean SD	Total	Factor 1	Factor2	Factor 3	Factor 4	Factor 5
Total ^a^	114.28 30.83	0.93	0.90	0.64	0.56	0.50	0.63
			<.0001	<.0001	<.0001	<.0001	<.0001
			0.90				
Factor 1	51.52 15.01			0.47	0.38	0.33	0.59
				<.0001	<.0001	<.0001	<.0001
				0.92			
Factor 2	15.25 9.64				0.13	0.14	0.20
					<.0001	<.0001	.0326
					0.97		
Factor 3	15.61 8.14					0.11	0.30
						.0008	<.0001
						0.89	
Factor 4	12.89 7.26						0.20
							<.0001
							0.80
Factor 5	19.01 4.67						–

a. Total scale excludes item pcosqd5

**Table 8:** Means, standard deviations, pearson correlations, and reliabilities for the total scale and six subscales of the PCOSQ-50 for six factors (*n* = 935)

Factors	Mean SD	Total	Factor 1	Factor2	Factor 3	Factor 4	Factor 5	Factor 6
Total	103.28 28.51	0.93	0.90	0.68	0.58	0.29	0.72	0.61
			<.0001	<.0001	<.0001	<.0001	<.0001	<.0001
			0.88					
Factor 1	41.56 13.71			0.50	0.35	0.33	0.63	0.51
				<.0001	<.0001	<.0001	<.0001	<.0001
				0.92				
Factor 2	15.25 9.64				0.13	0.14	0.29	0.19
					<.0001	<.0001	<.0001	<.0001
					0.97			
Factor 3	15.61 8.14					0.11	0.37	0.28
						.0008	<.0001	<.0001
						0.89		
Factor 4	12.89 7.26						0.23	0.19
							<.0001	<.0001
							0.84	
Factor 5	15.08 4.14							0.57
								<.0001
								0.78
Factor 6	15.78 4.11							–

**Table 9:** Means, standard deviations, pearson correlations, and reliabilities for the total scale and six subscales of the PCOSQ-50 for seven factors (*n* = 935)

Factors	Mean	SD	Total	Factor 1	Factor2	Factor 3	Factor 4	Factor 5	Factor 6	Factor 7
Total	99.08	27.84	0.93	0.56	0.64	0.87	0.52	0.67	0.64	0.51
				<.0001	<.0001	<.0001	<.0001	<.0001	<.0001	<.0001
				0.97						
Factor 1	15.61	8.14			0.12	0.34	0.11	0.36	0.26	0.25
					.0004	<.0001	.0008	<.0001	<.0001	<.0001
					0.93					
Factor 2	13.22	8.93				0.47	0.13	0.34	0.29	0.13
						<.0001	<.0001	<.0001	<.0001	<.0001
						0.85				
Factor 3	29.63	10.87					0.32	0.63	0.57	0.45
							<.0001	<.0001	<.0001	<.0001
							0.89			
Factor 4	12.89	7.26						0.24	0.25	0.16
								<.0001	<.0001	<.0001
								0.83	0.47	0.47
Factor 5	17.10	5.01							0.52	0.54
	14.68	4.17							<.0001	<.0001
									0.83	
Factor 6	13.05	3.49								0.32
										<.0001
										0.47
Factor 7										–

Based on the scree plot, model fit, use of all items, and no complex situations, the seven-factor model was identified as the best fit. Table 10 shows each factor, the number of items from the PCOSQ-50, and the chosen label for each factor.

**Table 10:** Chosen labels for each factor as identified by the 7-factor model

Factor	Item	Label	PCOSQ-50 Items
1	1	**Hirsutism**	In the past 4 weeks, how often have you felt concerned because of excess facial hair?
	2		In the past 4 weeks, how often have you felt embarrassed because of having excess facial hair?
	3		In the past 4 weeks, how often have you felt concerned about the progression of excess body and facial hair?
	4		In the past 4 weeks, how often have you felt embarrassed because of having excess body hair?
	5		In the past 4 weeks, how often have you felt concerned about rapid regrowth of unwanted hair after its removal?
	6		In the past 4 weeks, how often have you experienced the need to cover your body and/or face because of excess hair?
2	7	**Fertility**	In the past 4 weeks, how often have you experienced concern about infertility?
	8		In the past 4 weeks, how often have you felt concerned about infertility in the future?
	9		In the past 4 weeks, how often have you felt sad seeing pregnant women?
	10		In the past 4 weeks, how often have you felt you will accept all PCOS manifestations if assured of pregnancy?
	11		In the past 4 weeks, how often have you felt sad seeing children?
	12		In the past 4 weeks, how often have you felt fear of abortion?
	13		In the past 4 weeks, how often have you felt uselessness of sexual intercourse due to infertility?
3	14	**Isolation/Trepidation**	In the past 4 weeks, how often have you felt lack of satisfaction with being a woman?
	15		In the past 4 weeks, how often have you felt the desperate need for a cure for PCOS?
	16		In the past 4 weeks, how often have you felt a lack of family support and acceptance of PCOS?
	17		In the past 4 weeks, how often have you felt the need to complain with others about PCOS?
	18		In the past 4 weeks, how often have you felt different from other women without PCOS?
	19		In the past 4 weeks, how often have you felt difficulty communicating with others about PCOS?
	20		In the past 4 weeks, how often have you felt lack of satisfaction with your role as a spouse or partner?
	21		In the past 4 weeks, how often have you felt fear of diseases such as diabetes, hypertension, and heart disease?
	22		In the past 4 weeks, how often have you felt the urge to abandon treatment because of repetitive visits to doctors?
	23		In the past 4 weeks, how often have you experienced concern about the long-term effects of PCOS medication?
	24		In the past 4 weeks, how often have you experienced fear of cancer?
	25		In the past 4 weeks, how often have you experienced fear of divorce or separation?
	26		In the past 4 weeks, how often have felt concerned about menstruation at long intervals?
	27		In the past 4 weeks, have you felt concerned about the complete cessation of menstruation?
4	28	**Sexual Function**	In the past 4 weeks, how often have you experienced lack of sexual desire?
	29		In the past 4 weeks, how often have you experienced the lack of libido because of PCOS?
	30		In the past 4 weeks, how often have you experienced lack of stimulation?
	31		In the past 4 weeks, how often have you felt ashamed of sexual coldness or unresponsiveness?
	32		In the past 4 weeks, how often have you felt unsatisfied with sex?
	33		In the past 4 weeks, how often have you felt lack of lubrication during sexual intercourse?
	34		In the past 4 weeks, how often have you experienced lack of orgasm?
5	35	**Self-Esteem**	In the past 4 weeks, how often have you suffered from low self-esteem due to PCOS?
	36		In the past 4 weeks, how often have you felt ugly or unattractive?
	37		In the past 4 weeks, how often have you felt lack of satisfaction with your appearance (self-image)?
	38		In the past 4 weeks, how often have you suffered from embarrassment due to your appearance?
	39		In the past 4 weeks, how often have you experienced aggressiveness due to PCOS?
	40		In the past 4 weeks, how often have you felt different from women without PCOS?
6	41	**Emotional**	In the past 4 weeks, how often have you suffered from a bad mood due to PCOS?
	42		In the past 4 weeks, how often have you experienced trouble dealing with others?
	43		In the past 4 weeks, how often have you experienced lack of control of emotions?
	44		In the past 4 weeks, how often have you experienced impatience due to PCOS?
	45		In the past 4 weeks, how often have you felt pessimistic about the treatment for PCOS?
	46		In the past 4 weeks, how often have you felt the urge to abandon treatment because of repetitive visits to the doctor?
7	47	**Obesity**	In the past 4 weeks, how often have you felt willingness to reduce your weight to more attractive for your spouse or significant other?
	48		In the past 4 weeks, how often have you felt concerned being overweight?
	49		In the past 4 weeks, how often have you felt the need to decrease your weight to control PCOS status?
	50		In the past 4 weeks, how often have you felt concerned about a fast return to your previous weight after any weight loss?

**Fig. 3:**
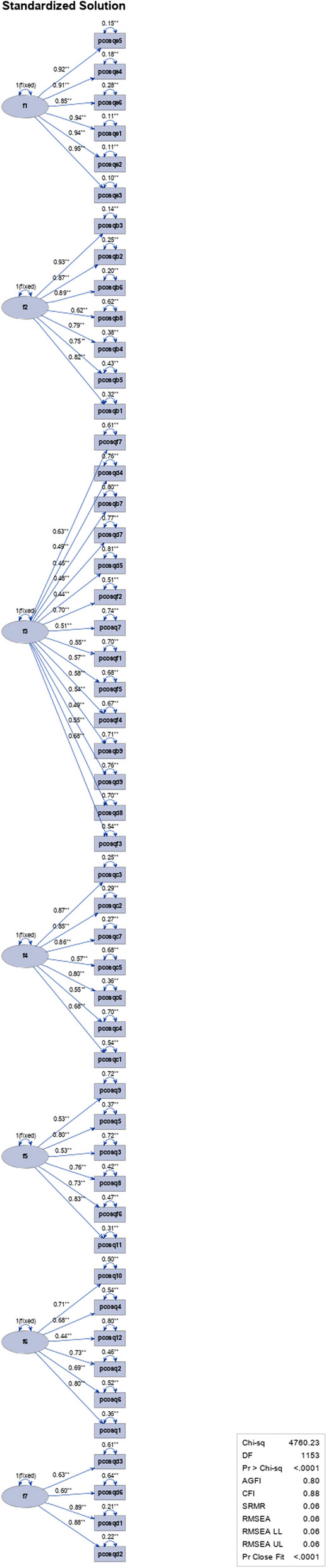
Path diagram for the confirmatory analysis of the 7-factor PCOSQ-50

Confirmatory factor analysis (CFA) was performed for the PCOSQ-50 on seven factors (Table 9). The goodness-of-fit for the model was assessed using chi-square statistics of 5547.78 with 1105-degree freedom (*P* < 0.0001), chi square ratio with degree freedom of 5, RMSEA and SRMSR of 0.06, the normed fit index or NFI of 0.82, the non-normed fit index or NNFI of 0.85, and CFI of 0.85. The results showed that the standardized factor loading was significant for all items for seven factors. The loading was from 0.45 to 0.74,0 .85 to 0.94, 0.62 to 0.92, 0.54 to 0.87, 0.44 to0 .70,0 .68 to 0.80, and 0.79 to 0.84 for factors 1 to 7 respectively. The R-Square was from 0.21 to 0.50, 0.72 to 0.90, 0.38 to 0.86, 0.30 to 0.75, 0.20 to 0.49, 0.46 to 0.64, and 0.63 to 0.71 for factors 1 to 7 respectively (see Fig. 3).

### Labeling the domains

The exploratory factor analysis revealed that a 7-factor model was a better fit than the current 6-factor model incorporated within the PCOSQ-50. The thematic analysis approach to labeling the seven factors or question groupings (henceforward called domains) yielded the following: *hirsutism*, *fertility, isolation/trepidation, sexual function, self-esteem, emotional*, and *obesity* (see Table 10). Three domain names remained unchanged as they were appropriately descriptive (*hirsutism, fertility, sexual function*); three domains were relabeled (*obesity, self-esteem, emotional*). An additional emergent domain was labeled (*trepidation/isolation*).

The current PCOSQ-50 has a domain comprised of nine questions labeled “Obesity and Menstrual Disorders.” However, only four questions were about obesity concerns; two were about menstrual disorders; two were about comorbidities of hypertension and cancer; and the remaining one was about repetitive visits to doctors. Therefore, exploratory factor analysis suggested developing *obesity* into a singular category and shifting the two menstrual questions to *fertility*. Our thematic analysis relabeled this domain by excluding “menstrual disorders” while retaining “obesity.” The remaining questions in this domain used words such as “fear,” and “lack of”, and explored social support issues (e.g.,” In the past 4 weeks, how often have you felt a lack of family support and acceptance of PCOS?” and “In the past 4 weeks, how often have you felt fear of diseases such as diabetes, hypertension, and heart disease?). Thematic analysis of this category resulted in a new label of *isolation/trepidation* The original PCOSQ-50 also has domains labeled “Psychosocial/Emotional” and “Coping,” which according to thematic analysis, were better described as two domains labeled “Self-Esteem” and “Emotional,” respectively.

## Discussion

Authors of the PCOSQ-50 last explored the factor structure in 2018. At that time, these authors called for further studies to better establish the validity and reliability of the PCOSQ-50 [11]. This call along with feedback from survey respondents [13] prompted this study, with the purpose to explore and validate the factor structure of the PCOSQ-50 using data from the largest cross-sectional PCOS sample to date (*n* = 935). Our team revealed that the PCOSQ-50, the more commonly used PCOS-specific HRQoL instrument, has good reliability but may be best described using a 7-factor model with the following labels: hirsutism, fertility, isolation/trepidation, sexual function, self-esteem, emotional, and obesity. Confirmatory analysis validated the 7-factor model.

Based on our results, the current version of the PCOSQ-50 may possibly misrepresent the impact of certain domains on HRQoL and/or misclassify PCOS-specific problems that impact HRQoL. For example, statements about menstrual irregularities are combined with statements about obesity and this subscale is titled, “Obesity and Menstrual Disorders.” According to the recommended Rotterdam criteria for PCOS diagnosis, four PCOS phenotypes exist, such that both lean and overweight/obese women may have PCOS. Thus, lean women with PCOS may have menstrual irregularities in the presence of hyperandrogenism [22] and overweight or obese women with PCOS may not always have menstrual irregularities [23]. As such, it would be difficult to distinguish the prevalence and impact of menstrual issues versus obesity concerns. The 7-factor model included the category of “obesity” as its own subscale, whereas the category of “menstrual disorders” appeared in a different domain.

The 7-factor model shifted questions among groupings, creating an additional factor. This new domain was labeled “Isolation/Trepidation.” Thematic analysis revealed that associated questions comprise words such as *fear* and *lack of* and express social support issues. This new category is consistent with the literature, as perceived loneliness is strongly associated with PCOS [24] and women with PCOS have routinely reported lack of social connection with others who understand them and/or PCOS [25, 26]. The label “trepidation” was chosen because it captures feelings such as anxiety, fear, and apprehension, all sentiments expressed in the questions and consistent with research evidence that women with PCOS are more likely to have *fear* about their future health, perceived loss of femininity, and infertility [27].

The original PCOSQ-50 also has the domains “Psychosocial/Emotional” and “Coping,” which according to the 7-factor model, was best described as two domains labeled “Self-Esteem” and “Emotional.” These labels are more descriptive and better differentiate two concerns among women with PCOS that have been shown to be mutually independent: low self-esteem and depression [28].

Whereas this was an international sample of women with PCOS, the sample originated mostly from the US. Cultural impact often necessitates cultural adaptation to HRQoL surveys, as the meaning of HRQoL and its components can be cultural-specific [29]. Therefore, the results reflect primarily Westernized culture, and cannot be generalized to cultures outside the US.

### Indications and future research

Recently, the technology app Flo, the most downloaded AI-driven menstrual and symptom tracker for women, was used to analyze the largest known PCOS symptom dataset to obtain a comprehensive understanding of the most prevalent and bothersome PCOS symptoms. Across five countries, women with PCOS aged 25–36 years frequently reported the following symptoms: bloating, hirsutism, irregular cycles, hyperpigmentation, and baldness [30]. Of these, three (bloating, hyperpigmentation, and baldness) are not mentioned within the PCOSQ-50. An ongoing debate concerns whether the full range of heterogenous PCOS symptoms should be incorporated into a PCOS-specific HRQoL instrument [6, 7].

In a cross-sectional study comparing the PCOSQ-50 and depressive symptoms scores between women with PCOS aged 18–42 years and those aged ≥43 years, HRQoL seemed to improve as women with PCOS aged however depressive symptomology remained moderately high [31]. The findings also indicated that obesity and hirsutism continued to negatively affect HRQoL, whereas menstrual factors were less of an issue, as > 75% of the participants identified themselves as menopausal with fertility issues resolved. An implication is that the PCOSQ-50 may be incomplete, especially when assessing peri-postmenopausal women with PCOS. Additionally, the PCOSQ-50 was developed and has only been psychometrically assessed using data collected from reproductive-aged women with PCOS. Thus, the PCOSQ-50 seems inappropriate for older women with PCOS, indicating a need for either a revision of the current instrument or the creation of a new one for different age parameters.

Lastly, cultural shifts over the last decade necessitate review of the current PCOSQ-50 for inclusive and person-centered language. For example, as the PCOSQ-50 is currently written, a traditional ideology of beauty and the female role is promoted and includes phrasing comparing women with PCOS to “normal” women. Such negative phrasing can unintentionally marginalize a single group of people and reinforce the sociocultural stigmatization of a group already at risk for stigma-related stress [25].

As a next step, the authors will conduct assessments of face and content validity to further assess the factor structure revealed by the exploratory factor and confirmatory analyses. We will conduct interviews with PCOS experts, including healthcare providers who treat women with PCOS and women with PCOS, inquiring about their perceptions of the PCOSQ-50. The PCOS experts will also be asked to assess content and face validity of the PCOSQ-50 using a content validity index and impact scores.

### Strengths and limitations

The survey data used for this factor analysis was from the largest cross-sectional study of women with PCOS aged 18–42 years to date. The survey was administered online, thus confirmation of PCOS diagnosis was not required and all answers were self-reported data. As such, responses were subject to recall and social desirability biases. To help prevent robotic responses, internet safeguards such as CAPTCHA were added. Facebook was used for its PCOS-specific pages, as users must pass an initial level of screening to participate on the page. In addition, the PCOSQ-50 was developed by Nasiri-Amiri and colleagues after conducting a mixed-method, sequential, exploratory study in 2011–2012 with 23 women diagnosed with PCOS aged 18–40 years [9]. The use of a primarily US-centered population was both a strength and limitation. First, until now, all testing of PCOS-specific HRQoL have been with women outside the US; thus, we have added a data from another cultural group. However, our results cannot be generalized to areas outside of the US.

## Conclusion

The purpose of this study was to explore the factor structure of the PCOSQ-50 using an international cross-sectional survey data from women with PCOS aged 18–42 years. Overall, the PCOSQ-50 demonstrates reliability when assessing the HRQoL among women with PCOS of reproductive age. However, the factor analysis yielded information that the domains may better assess the impact of different PCOS symptoms if separated into seven categories as opposed to the current six categories. Separating menstrual conditions from obesity concerns may more accurately reflect the prevalence of each and then the variation between PCOS phenotypes. As such, menstrual irregularities may not apply to peri-postmenopausal women with PCOS. More research is needed to adapt the current PCOSQ-50 for a mostly US population and to assess the PCOSQ-50 in other cultural populations outside the US. An age-appropriate PCOS-specific HRQoL instrument for older women with PCOS should be investigated and created. Lastly, further research could include a content analysis of the PCOSQ-50 from the perspective of peri-postmenopausal women with PCOS.

### Supplementary Information

**Additional file 1. **Geographic Areas Outside of the United States Represented in the Sample (*n* = 935).

## Data Availability

The dataset analyzed during the current study is available in the Open Science Framework at https://osf.io/654kr/.

## Abbreviations

CAPTCHA: Completely Automated Public Turing test to tell Computers and Humans Apart
EFA: Exploratory Factor Analysis
HRQoL: Health-related quality of life
MPCOSQ: Modified Polycystic Ovary Syndrome Questionnaire
PCOS: Polycystic ovary syndrome
PCOSQ-50: Polycystic Ovary Syndrome Questionnaire
REDCap: Research Electronic Data Capture
SD: Standard deviation
