# Supplementary material for: Exploratory factor and confirmatory analyses of the polycystic ovary syndrome health-related quality of life questionnaire (PCOSQ-50)
Source: Health Qual Life Outcomes. 2024 Feb 3;22:15. doi: 10.1186/s12955-024-02228-z (PMC10837866; doi:10.1186/s12955-024-02228-z)
Supplement: Supplementary file 1 — Additional file 1. Geographic Areas Outside of the United States Represented in the Sample (n = 935). [file 12955_2024_2228_MOESM1_ESM.docx]

**Additional File 1:**

*Geographic Areas Outside of the United States Represented in the Sample (n=935)*

| **LOCATION** | **# of Respondents**  **(n=935)** |
| --- | --- |
| Africa  Zimbabwe | 1  1 |
| Africa, East  Kenya  Uganda | 2  1 |
| Africa, South  Cape Town  Gauteng  Johannesburg | 2  2  2  1 |
| Africa, West  Nigeria | 4 |
| Asia | 2 |
| Asia, East  South Korea  Seoul | 1  1 |
| Asia, South  Pakistan | 4 |
| Asia, Southeast  Malaysia  Myanmar  Philippines  Singapore | 1  2  1  7  4 |
| Asia, Southwest  Saudi Arabia | 3 |
| Australia  Ballarat  Brisbane  New South Wales  Perth  Queensland  Sydney | 17  1  1  2  1  3  3 |
| Bahamas | 2 |
| Canada  Alberta  British Columbia  Nova Scotia  Ontario  Kitchener  Ottawa  Saskatchewan | 13  7  1  4  3  1  1  1 |
| Caribbean  Puerto Rico  Trinidad and Tobago | 2  1  2 |
| Egypt  Cairo | 1  1 |
| Europe  Iceland | 7  1 |
| Europe, Central  France  Germany  Poland | 2  2  1 |
| Europe, North  Finland  Norway | 1  3 |
| Europe, Southeast  Bosnia and Herzegovina  Croatia  Slovenia | 1  2  1 |
| Europe, West  Belgium | 1 |
| Isle of Man | 1 |
| Kingdom of Denmark  The Faroe Islands | 1 |
| Middle East  Qatar | 2  1 |
| United Arab Emirates  Dubai | 2  1 |
| United Kingdom  England  Cheshire  Coventry  Leeds  Peterborough  Plymouth  West Yorkshire  Ireland  Scotland  Buckie Morayshire  Glasgow  Wales | 18  11  1  1  1  1  1  1  5  2  1  1  3 |
| **TOTALS** | 187 |
| **Percentage** | 20% |
